# Presence of ROS in Inflammatory Environment of Peri-Implantitis Tissue: In Vitro and In Vivo Human Evidence

**DOI:** 10.3390/jcm9010038

**Published:** 2019-12-23

**Authors:** Eitan Mijiritsky, Letizia Ferroni, Chiara Gardin, Oren Peleg, Alper Gultekin, Alper Saglanmak, Lucia Gemma Delogu, Dinko Mitrecic, Adriano Piattelli, Marco Tatullo, Barbara Zavan

**Affiliations:** 1 Head and Neck Maxillofacial Surgery, Department of Otoryngology, Tel-Aviv Sourasky Medical Center, Sackler Faculty of Medicine, Tel-Aviv University, Weizmann street 6, 6423906 Tel-Aviv, Israel; mijiritsky@bezeqint.net; 2 Maria Cecilia Hospital, GVM Care & Research, Cotignola, 48033 Ravenna, Italy; Letizia.ferroni@gmail.it (L.F.); chiara.gardin@gmail.it (C.G.); 3 Department of Otolaryngology Head and Neck Surgery and Maxillofacial Surgery, Tel-Aviv Sourasky Medical Center, Sackler School of Medicine, Tel Aviv University, 701990 Tel-Aviv, Israel; orenpeleg@gmail.com; 4 Istanbul University, Faculty of Dentistry, Department of Oral Implantology, 34093 Istanbul, Turkey; alpergultekin@hotmail.com (A.G.); alper.saglanmak@istanbul.edu.tr (A.S.); 5 University of Padova, DpT Biomedical Sciences, 35133 Padova, Italy; Lucia.Gemma.delogu@unipd.it; 6 School of Medicine, Croatian Institute for Brain Research, University of Zagreb, Šalata 12, 10 000 Zagreb, Croatia; dinko.mitrecic@mef.hr; 7 Department of Medical, Oral, and Biotechnological Sciences, University of Chieti-Pescara, via dei Vestini 31, 66100 Chieti, Italy; piateli@gmail.com; 8 Marrelli Health-Tecnologica Research Institute, Biomedical Section, Street E. Fermi, 88900 Crotone, Italy; marco.tatullo@tecnologicasrl.com; 9 Department of Medical Sciences, University of Ferrara, via Fossato di Mortara 70, 44123 Ferara, Italy

**Keywords:** Peri-implantitis, histological analysis, immunohistochemical analyses

## Abstract

Analyses of composition, distribution of cellular and extracellular matrix components, and molecular analysis of mitochondria related genes of bone loss in the presence of inflammatory environment in humans was the aim of the present project. As a human model we chose peri-implantitis. Morphological analyses were performed by means classical histological, immunohistochemical, and SEM (scanning electron miscroscopy) test. Gene expression analysis was performed to evaluate epithelium maturation, collagen fiber production, and genes related to mitochondrial activity. It was found that a well-defined keratinocyte epithelium was present on the top of all specimens; a distinct basal lamina was present, as well as desmosomes and autophagic processes related to the maturation of keratinocytes. Under this epithelium, a full inflammatory cell infiltrate was present for about 60% of the represented by plasma cells. Collagen type I fibers were present mainly in the form of fragmented cord tissue without cells. A different distribution of blood vessels was also present from the apical to the most coronal portion of the specimens. High levels of genes related to oxidative stress were present, as well as the activation of genes related to the loss of ability of osteogenic commitment of Mesenchymal stem cells into osteoblasts. Our study suggests that peri-implantitis lesions exhibit a well defined biological organization not only in terms of inflammatory cells but also on vessel and extracellular matrix components even if no difference in the epithelium is evident, and that the presence of reactive oxygen species (ROS) related to the inflammatory environment influences the correct commitment of Mesenchymal stem cells.

## 1. Introduction

Inflammation is the immune response i to foreign pathogens. If the system is prolonged or inefficient, it changes to the chronic state of inflammation often associated with loss of tissues such as bone. During this stage, high levels of ROS are present in the tissue since it acts as both a signaling molecule and a mediator of inflammation. Their high level is often harmful to cells because they oxidize lipid cellular constituents and protein, damaging the DNA. At the protein level, PKCs (protein kinase C) protein are influenced by ROS since PKC isoforms contain redox-sensitive cysteine residues in both regulatory and catalytic domains. Indeed, in our previous work, we demonstrated that activation of PKC beta by reactive oxygen species (ROS) dependent manners induces the commitment of Mesenchymal stem cells (MSC) into adipocytes [1,2]. In chronically inflamed bone tissue is not rare to find adipocytes instead of osteocytes causing ROS bone loss. In light of this consideration, in the present study we analyzed a human bone loss model related to a chronic state of inflammation: peri-implantitis. By definition, peri-implantitis, according to the current literature [3], is implant sites with clinical signs of inflammations, bleeding on probing, and either probing socket depths 3–5 mm or radiographic proven bone loss or both. Several studies suggest that peri-implant disease is a complex condition, and, in this light, it needs to be thoroughly investigated [4,5,6,7,8,9,10,11,12,13,14,15]. To this aim, in this work we focused our attention on ROS physiology in severe peri-implantitis.

## 2. Materials and Methods 

### 2.1. Patients

This is a cross-sectional study, where 40 non-smoker individuals, aged 30–50 years old, were recruited: 20 patients selected with peri-implantitis and 20 individual healthly patients (control group). The subjects were recruited among patients who had attended the School of Dental Medicine of Tel-Aviv University Ramat Aviv (Israel), and the University. All individuals had provided informed consent to be involved in the study, and the study was conducted in compliance with the Declaration of Helsinki ethical guidelines [16].

The study used a convenience sample because it was designed as a hypothesis generator. The exclusion criteria were as follows: ongoing orthodontic therapy; systemic disorders; drug administration during the past three months; pregnancy; history of bisphosphonates, monoclonal antibodies, high dosage corticosteroids therapy, or radiotherapy of the cervicofacial district.

The inclusion criteria for the group selected by peri-implantitis were as follows: presence of one or more implants with a minimum loading period of 12 months; peri-implant probing pocket depth >5 mm; peri-implant presence of bleeding on probing (with/without suppuration); radiographic signs of crestal bone loss in at least one area around an implant; and exposure of at least two implant threads. The control group consisted of subjects undergoing dental implant positioning who had no history or clinical signs of periodontitis or peri-implantitis; had a probing pocket depth equal to or less than 4 mm; and had no radiographic signs of peri-implant bone resorption.

In the test group, circumferential peri-implant soft tissue samples were collected during resective surgical treatment of peri-implantitis or in case of extraction of failed implants due to peri-implant disease. In the control group, semi-submerged healing was chosen and the retrieved specimen of mucosa, allowing the positioning of a healing abutment of larger diameter at the second stage after 8–12 weeks from implant placement, was collected instead of being discharged as waste material as usual.

### 2.2. Immunohistochemistry and Histomorpholgical Analyses

For histological analyses, specimens were treated with formalin, paraffin-embedded, and stained with hematoxylin and eosin, collagen type I (Coll-1, Abcam, Cambridge, UK); fibers, CD31 for endothelial cells (Abcam, Cambridge, UK), keratin antibody (anti keratine 8 antibody, Abcam, Cambridge, UK), and for the visualization, treated with acid phosphatase anti-acid phosphatase. Briefly, after non-specific antigen sites were saturated with 1/20 serum in 0.05 M maleate TRIZMA (Abcam, Cambridge, UK; pH 7.6) for 20 s, 1/100 primary monoclonal anti-human Ab (Abcam, Cambridge, UK) was added to the samples. After incubation, immunofluorescence staining was performed with APAAP (anti-rabbit, Abcam, Cambridge, UK) secondary antibody. For histomorphological semi quantitative analyses, biopsies were fixed in 4% paraformaldehyde (Sigma–Aldrich, St. Louis, MO, USA) in phosphate-buffered saline (PBS, EuroClone, Milan, Italy) for 24 h, and then dehydrated in graded ethanol. After a brief rinse in xylene (Sigma–Aldrich), the samples were paraffin-embedded and cut into 5-μm-thick sections. Sections were then stained with the nuclear dye hematoxylin (Abcam, Cambridge, UK) and the counterstain eosin (Abcam, Cambridge, UK). In order to analyze the cellular response of cells to treatments, masked microscopic examinations by two researchers were performed, as previously described [17]. Briefly, 3 slides for each sample were analyzed by light microscopy, using 20× as the initial magnification. Each slide contained 3 sections of specimen, and 5 fields were analyzed for each tissue section. A semi quantitative analysis of the presence of the following cell types to compare Group A and Group B was used: (i) polymorphic nuclear cells (cells characterized by a nucleus lobed into segments and cytoplasmic granules, i.e., granulocytes); (ii) phagocytic cells (large mononuclear cells, i.e., macrophages and monocyte-derived giant cells); (iii) nonphagocytic cells (small mononuclear cells, i.e., lymphocytes, plasma cells and mast cells.); (iv) fibroblasts; (v) endothelial cells; (vi) keratinocyte; (vii) collagen fibers. All of these items were evaluated blindly and scored as absent (0), scarcely present (1–5 element x section), present (6–10 element x section), and abundantly present (11 over x section element). Experiments were performed at least three times and values were expressed as mean ± SD.

### 2.3. Immunofluorescence Staining

Slides were fixed in 4% paraformaldehyde in PBS for 10 min, and then incubated in 2% bovine serum albumin (BSA, Abcam, Cambridge, UK) in PBS for 30 min at room temperature. Samples were then incubated with primary antibodies in 2% BSA solution in a humidified chamber for 12 h at 4 °C. Samples were incubated with the primary antibodies (rabbit polyclonal anti-human LC3B, Abcam, Cambridge, UK, A0082, Dako, Milan, Italy). Immunofluorescence staining was performed using the secondary antibodies DyLight 549-labeled anti-rabbit IgG (H+ L) (KPL, Gaithersburg, MD, USA), and DyLight 488-labeled anti-mouse IgG (KPL) diluted 1:1000 in 2% BSA for 1 h at room temperature. Nuclear staining was performed with 2 μg/mL Hoechst H33342 (Sigma–Aldrich) solution for 2 min.

### 2.4. Transmission Electron Microscopy (TEM)

The samples were analyzed by TEM (Electronic Microscopy Service, Department of Biology, University of Padova, Padova, Italy). Briefly, samples were fixed in 2.5% glutaraldehyde in 0.1 M phosphate buffer pH 7.4 for 3 h, post-fixed with 1% osmium tetroxide, dehydrated in a graded series of ethanol, and embedded in araldite. Semithin sections were stained with toluidine blue and used for light microscopy analysis. Ultrathin sections were stained with uranyl acetate and lead citrate and analyzed with a Philips EM400 electron microscope. 

### 2.5. RT2 Profiler PCR Array

Total RNA was extracted with RNeasy Mini Kit (Qiagen GmbH, Hilden, Germany). 

The first-strand cDNA has been synthesized starting from 800 ng of total RNA from each sample (Qiagen Sciences, Germantown, MD, USA). Real-time PCR was performed according to the user manual of the Human Wound Healing RT2 Profiler PCR array (SABiosciences, Frederick, MD, USA) using RT2 SYBR Green ROX FAST Mastermix (SABiosciences). The results are reported as the ratio of genes in the peri-implantitis with control samples.

Briefly, primers and probes were selected for each target gene using Primer3 software. Gene expression was measured using real-time quantitative PCR on a Rotor-Gene 3500 (Corbett Research, Sydney, Australia). PCRs were carried out using the designed primers at 300 nm and SYBR Green I dye (Invitrogen, Carlsbad, CA) (using 2 mm MgCl_2_) with 40 cycles of 15 s at 95 uC and 1 min at 60 uC. All cDNA samples were analyzed in duplicate. The cycle threshold (Ct) was determined automatically by the software. The amplification efficiencies of the studied genes were 92 to 110%. For each cDNA sample, the Ct of the reference gene, L30, was subtracted from the Ct of the target sequence to obtain the DCt. The expression level was then calculated as 2DCt and expressed as the mean 6 SD of quadruplicate samples of two separate runs. The baseline is the expression in healthy tissue. 

### 2.6. Data Analysis

One-way ANOVA was used for data analysis. Levene’s test was used to demonstrate equal variance in the variables. Repeated-measures ANOVA with Bonferroni’s multiple comparison post hoc analysis was performed. T-tests were used to determine significant differences (*p* < 0.05). Reproducibility was calculated as the standard deviation of the difference between measurements. All testing was performed using SPSS software, version 16.0 (SPSS, Inc., Chicago, IL, USA; licensed by the University of Ferrara, Italy). The study used a convenience sample because it was designed as a hypothesis generator. The sample size of each test was driven by practical reasons (including costs, time, staff workload, and resources).

## 3. Results

**Figure 1:**
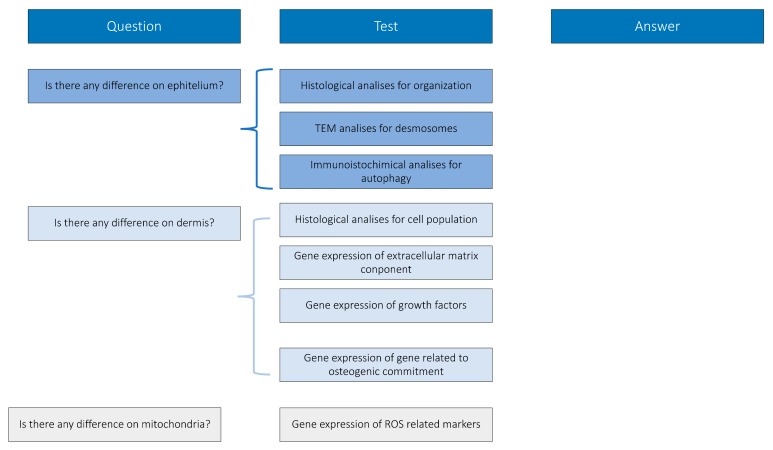
Experimental design.

Peri-implantitic tissues have been treated and analyzed following the experimental design reported in Figure 1, constructed in order to give biological answers to defined questions.

**Figure 2:**
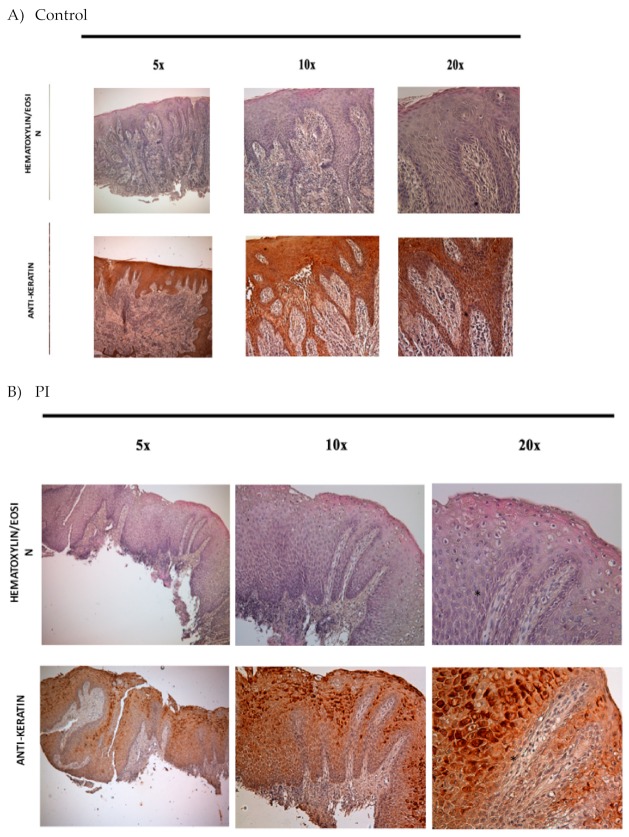

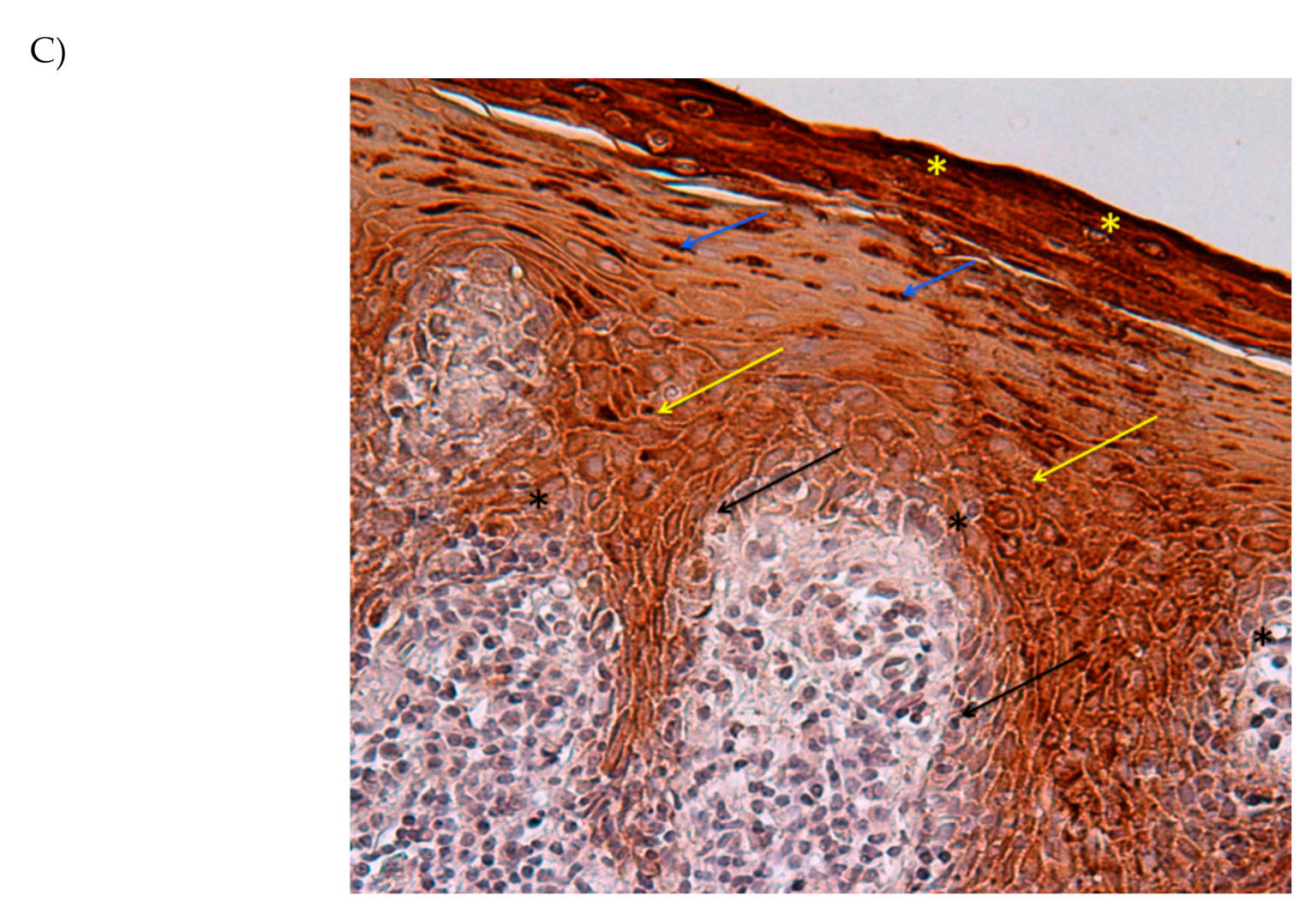
Histological staining with haematoxillin eosin and immunohistochemical analyses against keratin (brown) in control (**a**) and Peri-implantitic (PI) (**b**,**c**) samples. Black asterisks: cuboid keratinocytes (stratum germinativum), yellow arrows: cells of the upper spinous layers contain lamellar granules, blue arrows: stratum granulosum, yellow asterisks: stratum corneum, black arrows: basal lamina, between epidermis and dermis (100×).

Peri-implantitic (PI) tissues were analyzed by their morphological organization. As reported in Figure 2b, all the samples show the presence of two defined compartments: the epithelium and the dermis.

### 3.1. Epithelium

**Figure 3:**
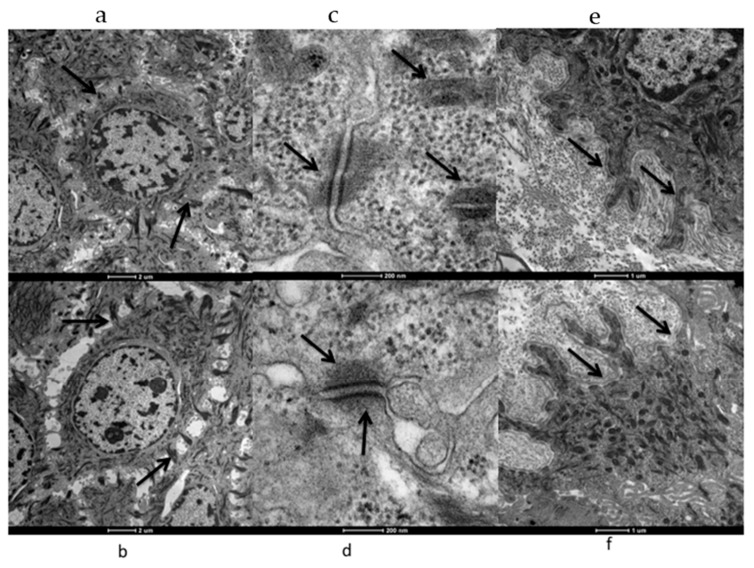
Transmission electron microscopy (TEM) analyses of keratinocytes interaction (black arrows **a**,**b**), desmosomal junction (black arrows **c**,**d**); basal lamina (black arrows **e**,**f**) in control (**a**,**b**,**c**) and PI samples (**d**,**e**,**f**).

Epithelium organization in the peri-implantitis specimens was evaluated by means of immunohistochemistry against keratin (Figure 2a for the control (healthy specimens; back for PI specimens). Figure 2c reveals that the epithelium is organized like a well epidermis tissue. The four typical layers were indeed present: stratum germinativum with the basal cell, the stratum spinosum with squamous cell, the stratum with granular cells, and the stratum corneum enrich with the corny end or horny cell. Black asterisks indicate the presence of cuboid keratinocytes that are present in stratum germinativum. Over this it is present a stratum of 8–12 squamous cells that form the stratum spinosum. Polyhedral in shape cells are moreover evident with a rounded nucleus (black arrows) forming the supra basal spinous cells. The upper spinous layers are well represented by cells larger in size, containing lamellar granules (yellow arrows). In the end, the supercoil layer is evident thanks to the presence of flattened cells with a cytoplasm enriched with abundant granules forming stratum granulosum (blue arrows). On the top of the epithelium the stratum corneum is well evident thanks the presence of the large, polyhedral-shaped horny cells with no nuclei (yellow asterisks). Cell adhesion proteins between keratinocytes on both healthy and PI tissue are presents and well developed and distributed. No difference between control and PI tissues was present (Figure 3a,b).

Electron microscopy analyses were more focused on desmosomes in control and PI samples. Desmosomes, intercellular junctions, mediate not only cell–cell adhesion but they anchor the cytoskeleton to the plasma membrane. With this structure, cells have mechanical support for tissues. In our samples it was well evident (Figure 3c,d) that desmosomal proteins regulate adhesion in healthy skin and in PI ephitelim and are equally present. The demarcation line, represented by basal lamina, between epidermis and dermis was also clearly evident (black arrows, Figure 3e,f) in both samples. 

**Figure 4:**
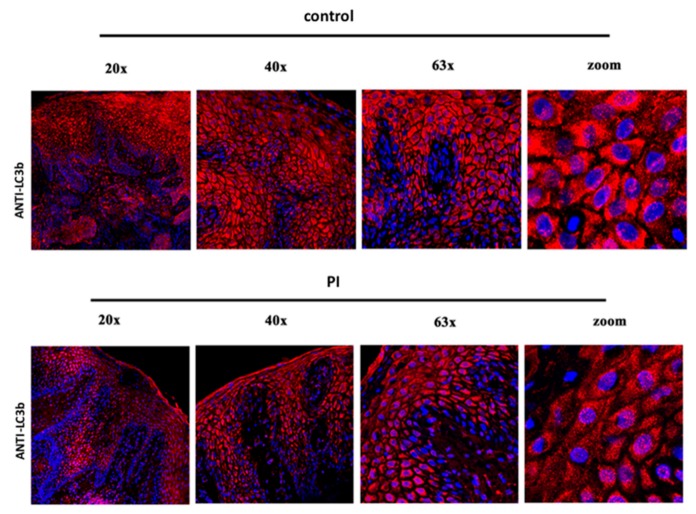
Immunofluorescence against autophagy processes in keratinocytes in control and PI samples. In all samples, positivity (in red) confirms the activation of the autophagy process during maturation of keratinocytes.

The health of epidermis was evaluated by means the analysis of the homeostasis of the keratinocytes. In healthy tissue (control Figure 4), the presence of autophagic proteins such as LC3b was reveled in keratinocyte layers, and the same presence with the same pattern of distribution was observed in PI keratinocytes, confirming that this aspect was conserving in both samples.

### 3.2. Inflamed Connective Tissue (ICT)

**Figure 5:**
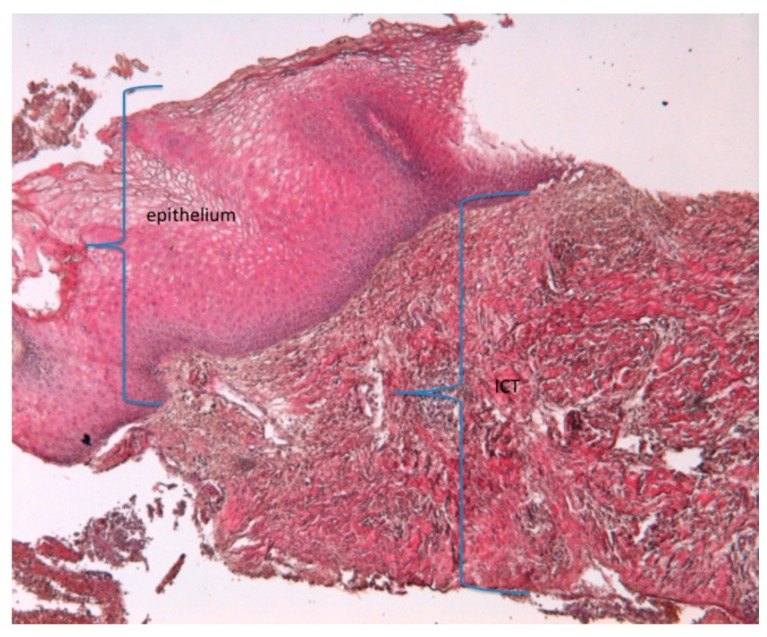
A histological staining with haematoxylin eosin. Presence of epithelium and ICT. (magnification 20×); ICT, inflamed connective tissue.

Beneath the epithelial layer, the dermis was not organized and showed the morphology of an inflammatory connective tissue (ICT) (blue bracket) (Figure 5).

It was well evident that a large area of this ICT portion was occupied by plasma cells, PMN (polymorphonucleate) cells, mostly formed by neutrophilic granulocytes. The composition of ICT could be resolved as:

- 45% plasma cells (macrophages 8% (large mononuclear cell); lymphocytes 7% (small mononuclear cells); plasma cells 48%; PMN 6%, mainly neutrophil granulocytes; residual tissue 31%)
- 15% collagen
- 12% vascular structure
- 28% other (8% fibroblasts)

Note that inflammatory cells could be moreover classified in phagocytic cells and non-phagocytic cells. In this tissue the primary composition of cells was formed by non phagocytic one. 

**Figure 6:**
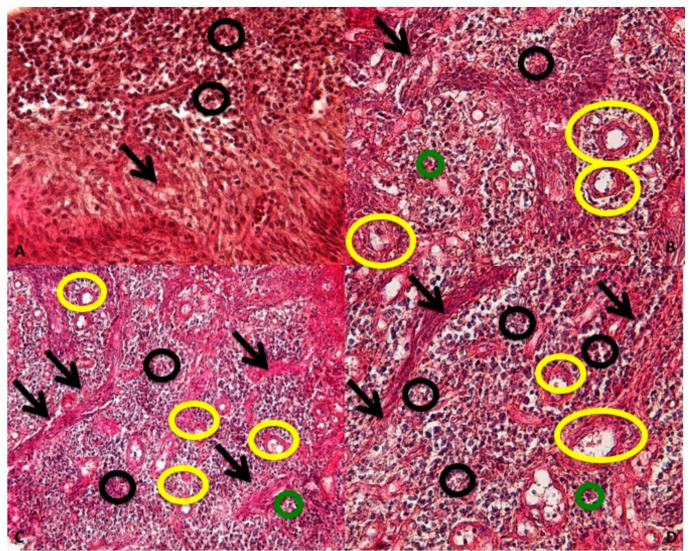
ICT organization: collagen fibers (black arrows); vascular structure (yellow circles); inflammatory cells (black and green circles). (Magnification 40×).

The ICT is about of 60% of the connective tissue present in the underlying dermis and it is composed of ICT contain collagen fibers (black arrows); vascular structure (yellow circles); and inflammatory cells (black and green circles) (Figure 6).

**Figure 7:**
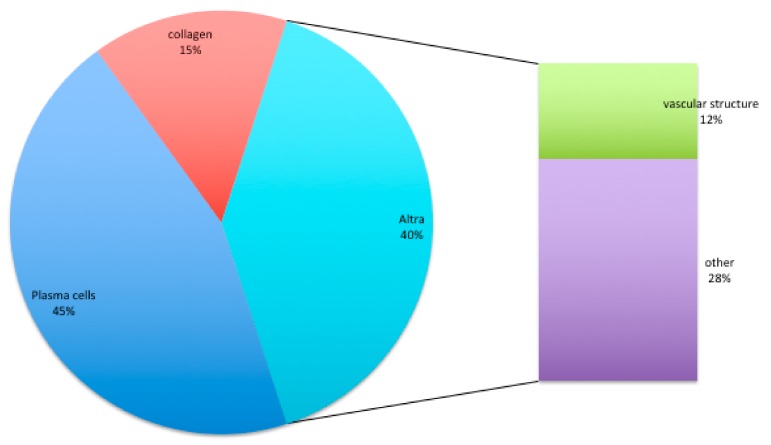
Composition of ICT tissue in % obtained by means of istomorphology.

Composition of ICT tissue (Figure 7):

- 45% plasma cells (macrophages 8% (large mononuclear cell); lymphocytes 7% (small mononuclear cells); plasma cells 48%; PMN 6% (cells characterized by a nucleus lobed into segments and cytoplasmic granules, i.e., granulocytes), mainly neutrophil granulocytes; residual tissue 31%)
- 15% collagen
- 12% vascular structure
- 28% other (8% fibroblasts)

**Figure 8:**
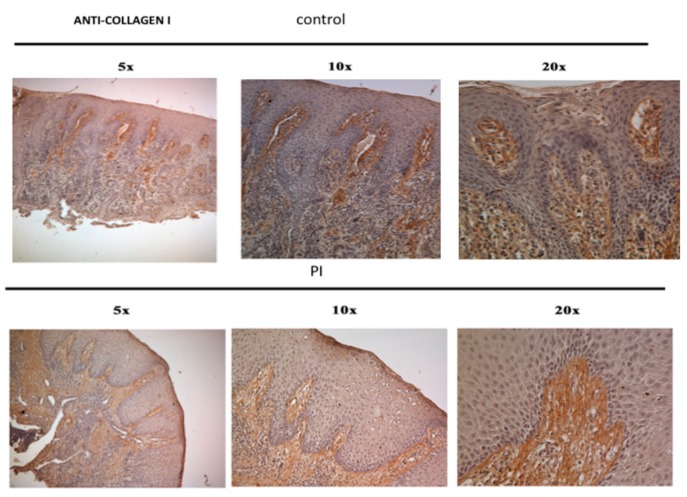
Collagen fibers present in the ICT and control samples.

Collagen fibers in PI samples (Figure 8) were often free of cells (black arrows). In the central and apical portion of the ICT, collagen fibers were indeed deprived of fibroblasts while inflammatory cells dominated the lesion (Figure 8).

**Figure 9:**
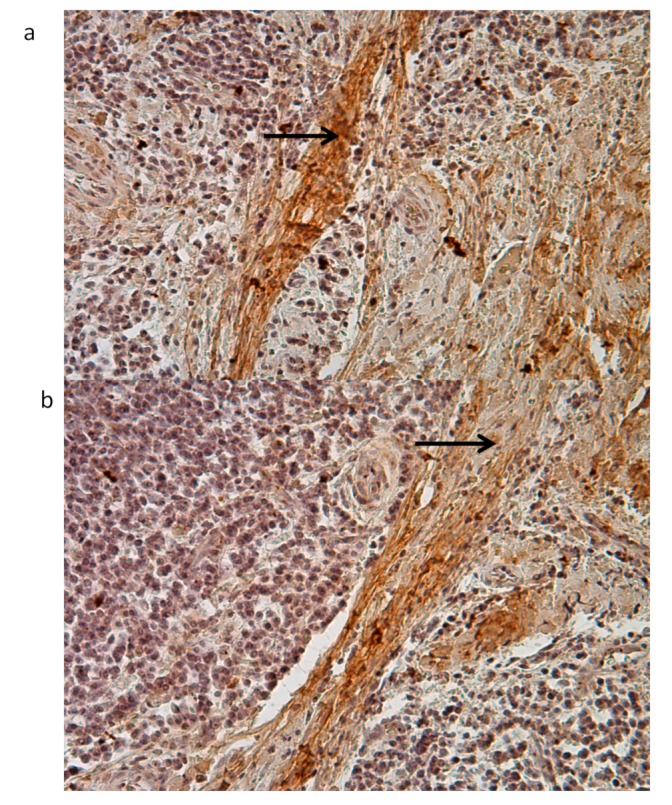
Immunohistochemistry against collagen fibers in PI samples (black arrows) (20×). Collagen fibers in PI samples (**a**,**b**) were often free of cell (black arrows).

Collagen fibers in PI samples (Figure 9a,b) were often free of cell (black arrows). In the central and apical portion of the ICT, collagen fibers were indeed deprived of fibroblasts while inflammatory cells dominated the lesion (Figure 9). 

Presence of an increase in growth factor gene expression revealed the predominance of colony stimulating factor 2 (CSF2) and CSF3 and of FGF (Fibroblast growth factor) family members.

**Figure 10:**
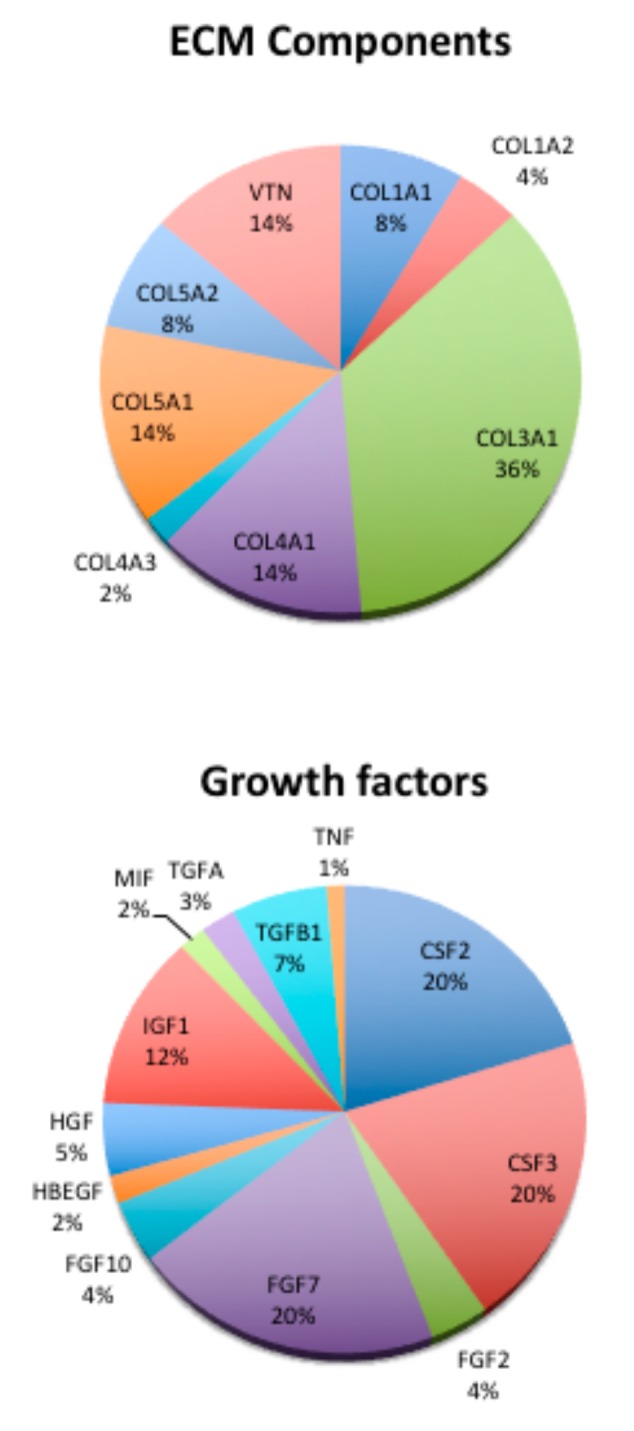
Composition of ICT tissue. Results are reported as % of gene expression of the genes in PI samples compare to the gene expression of housekeeping genes.

Gene expression related to the ECM components has been performed and reported as % increase of the value in PI compared to some genes in control tissues (Figure 10). The most representative components of ECM on PI tissues is collagen type III (COL3A1) with 36% increased value, followed by collagen type IV and V. The lowest collagen present is collagen type I. High levels of vitronectin (VNT) are also present.

Presence of an increase in growth factor gene expression, revealed the predominance of colony stimulating factor 2 (CSF2) and CSF3 and of FGF family members (FGF)2, FGF7, and FGF10. Transforming growth factors were more over present with alpha (TGFA) and beta 1 (TGFB1). 

**Figure 11:**
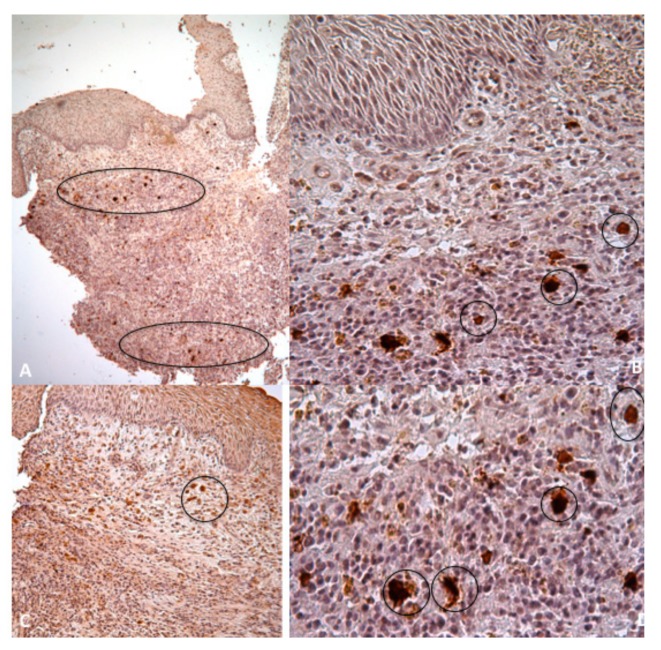
Immunohistochemistry against CD31 for endothelial cells show that the apical zone contains larger amounts of small vascular structures than the corresponding lower situation (black circles, A,C 5×; B,D 20×).

Large vascular units occupied the central part of the samples. The apical zone contains larger amounts of small vascular structures than present into the lower situation (Figure 11, black circles) 

### 3.3. Mitochondrial

**Figure 12:**
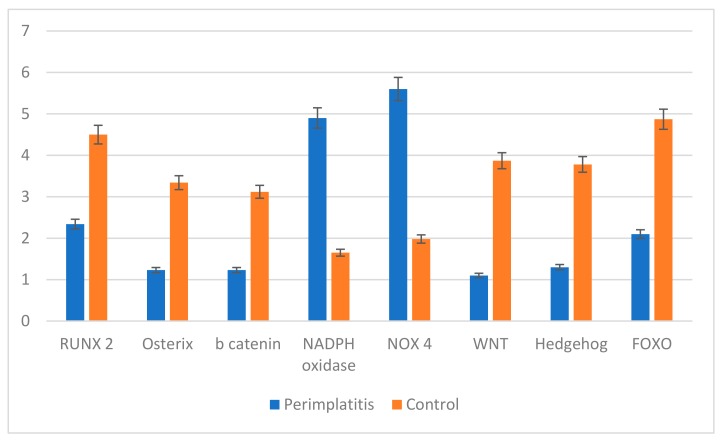
Gene expression of genes related to osteogenesis (RUNX, Osterix, b Catenin); RO production (MADPH oxidase; NOX4); pathway related to osteogenesis (WNT, HEDGEHOG, FOXO).

Gene expression analysis related to osteogenesis, ROS production, and pathways related to adipogenesis was performed. As reported in Figure 12, genes related to osteogenesis such as RUNX, Osterix, and b Catenin were down regulated in perimplantitis while in the control sample they were up regulated, supporting the presence of an osteogenic process. ROS production such as NADPH oxidase and NOX4 was up regulated in peri-implantitis and down regulated in control samples. The opposite trend was observed for the pathways related to osteogenesis: WNT, HEDGEHOG, and FOXO were down expressed in peri-implantitis and up regulated in the control.

## 4. Discussion

Bone loss is the injuries of the musculoskeletal system that induce bone fracture and, in odontoiatric field, when it occurs around the implants induces loss of osteointegration, inducing the well know phenomenon call peri-implantitis. The outcome of bone-healing is related to a number of factors and often the treatments do not induce to the optimal final step: good regeneration. In this view, the knowledge of the complexity of the physiological process of bone healing is fundamental for the optimal bone treatment. However, most of the knowledge of bone loss and bone healing is based on studies on animal models and, even if the biology seems to be similar, new biomechanical approaches are needed to be explored in humans with the aim to improve the healing outcome. 

Bone healing follows a specific course: inflammation, repair, and remodeling. Inflammation is often associated with vasodilatation and exudation of plasma and leukocytes. The tissue is characterized by hypoxia and low pH, blood-derived inflammatory cells, and proinflammatory or anti-inflammatory cytokines. In the context of inflammatory cells, the first cells recruited are the neutrophils that usually are attracted by dead cells and debris. They secrete proinflammatory cytokines such as IL-1, and 6, TNF, and TGF-β. Moreover, they should be present in order to improve revascularization. Usually this inflammatory event occurs over the first days and maximum levels of inflammatory cytochynes are within the first day. In several cases, a perturbation of the biology of the bone could induce chronic systemic inflammation close to the bone loss. In light of such consideration we choose as a human model of bone loss related to chronic inflammation peri-implantitis. As a first test we analyzed cell composition of the inflamed tissue, focusing our attention on the PMN population formed in prevalence by neutrophils. Neutrophils, which constitute of the first line of the innate immunity in humans, employ different approaches to kill and trap microorganisms. Moreover, it is well known that generation of reactive oxygen (ROS) is crucial for their recruitment and life cycle. The presence of an excessive amount of ROS could induce a detrimental effect on the extracellular matrix environment of host tissues. Indeed, markers of oxidative stress are detectable in many diseases. The redox state changes are related to the bone remodeling process which allows the continuous bone regeneration. Indeed, oxidative stress alters the bone remodeling process causing an unbalance between osteoclast and osteoblast activity since it activates the differentiation of pre-osteoclasts in osteoclasts and strengthens the bone, and ROS induces the apoptosis of osteoblasts and osteocytes, cells localized in the bone. Moreover ROS elicits a spectrum of responses ranging from proliferation, growth, and differentiation arrest, to cell death by activating numerous signaling pathways. 

Until now, no evidence of this has ever been detected on peri-implantitis. In our samples we found a great quantity of ROS and high gene expression levels of mitochondrial genes related to ROS production, confirming the abnormal presences of this marker in the peri-implantitic tissue. Moreover, in recent years researchers highlighted its role as signaling molecules [17,18,19,20] impacting MSC differentiation. Evidence supports the strong relation with impaired skeletal integrity: increased levels of intracellular ROS induce the MSC potential in osteogenic differentiation [21,22,23,24,25,26,27,28]. Our data support the evidence that bone loss in an inflammatory environment such as the one present in peri-implantitis could be due to a deregulated process of MSC into osteoblast related to ROS production.

In the end we focused our attention on another important factor affecting neutrophils: the composition of the extracellular matrix (ECM). Recently researchers [29,30,31,32,33] confirmed that ECM proteins such as fibronectin, laminin, collagen, and fibrinogen in inflammation play a pivotal role in signals regulating neutrophil recruitment and transmigration and proteolytic activity. This induces the production and release of oxidants that result from the interaction of neutrophils with ECM with the final aim to induce the remodeling of the local microenvironment directly to facilitate the access of other immune derived cells. This new environment must be closer to a soft tissue compare to the hard tissue such bone, so the final environment should be fibrotic and not more calcified: in the end bone loss occurs with lots of osseointegration. In this view we analysed the composition of the extracellular matrix. Our data showed disordered collagen fibrils that were moreover small and hypertrophic and demineralized. 

**Figure 13:**
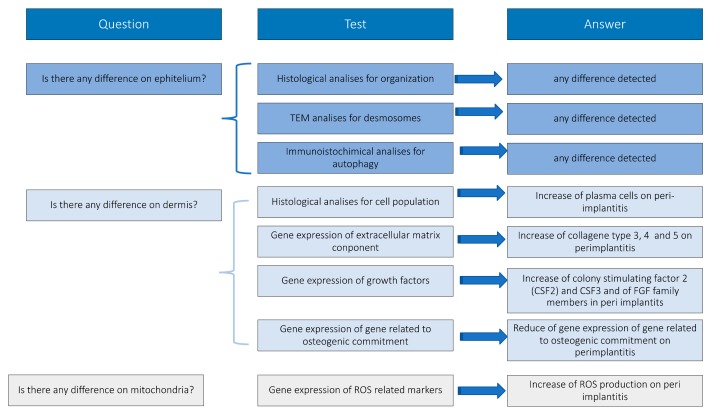
Conclusion.

In conclusion, the present study, following the results reported in Figure 13, in which they are organized in order to answer to our preliminary question, confirms that:

(1) In the peri-implantitis specimens, the epithelium is a well-defined tissue organized with compartments with a demarcation line between epidermis and dermis clearly evident.
(2) Beneath the epithelial layer, the dermis is not organized and shows the morphology of an inflamed tissue (ICT).
(3) The ICT is about of 60% of the connective tissue present in the underlying dermis
(4) The marginal portion of the ICT has large numbers of collagen fibers occurring together with several lymphocytes and plasma cells.
(5) Collagen fibers are parallel to the line of the epithelium and often free of the cell.
(6) Inflammatory cells mostly occupy the lesion and are formed by neutrophilic granulocytes;
(7) Abnormal levels of a high concentration of ROS are present in the lesion and they induce an alternated activation of neutrophils and the reduction of osteogenic commitment of MSC.

## 5. Conclusions

In the present work we have performed morphological analyses of peri-implantitic tissue. As reported by Dreyer et al. [1] the median prevalence of peri-implantitis indicates that dental implants are a successful treatment option for prosthetic rehabilitation in the general population. Based on a medium and medium-high level of evidence, smoking (effect summary OR 1.7, 95% CI 1.25–2.3) and diabetes mellitus (effect summary OR 2.5; 95% CI 1.4–4.5) are characterized by high levels of ROS production related to a general uncontrolled inflammatory situation (i.e., on the lung from the smokers) and in this article we confirm the high level of ROS could be strongly related to bone loss. Even if no data obtained from this study could help us in an early diagnosis of peri-implantitis or in definition about the biological event that could explain this degenerative process, the new results, related to the organization of the collagen cords, and organization of the epithelial layers that does not change from healthy compared to PI tissues, could open novel interpretations related to the relationship between the induction correlated to the extracellular matrix and the homeostasis of the cells around the implants. We agree that the overall amount of evidence to answer to our question is weak and future studies of a prospective, randomized, and controlled type, including sufficient sample sizes, are needed. The application of consistent diagnostic criteria is particularly important.
